# Therapeutic Approach of KRAS Mutant Tumours by the Combination of Pharmacologic Ascorbate and Chloroquine

**DOI:** 10.3390/biom11050652

**Published:** 2021-04-28

**Authors:** Orsolya Kapuy, Kinga Makk-Merczel, András Szarka

**Affiliations:** 1 Department of Molecular Biology, Institute of Biochemistry and Molecular Biology, Semmelweis University, H-1428 Budapest, Hungary; kapuy.orsolya@med.semmelweis-univ.hu; 2 Laboratory of Biochemistry and Molecular Biology, Department of Applied Biotechnology and Food Science, Budapest University of Technology and Economics, H-1111 Budapest, Hungary; makkmerczel@mail.bme.hu; 3 Biotechnology Model Laboratory, Faculty of Chemical Technology and Biotechnology, Budapest University of Technology and Economics, Szent Gellért tér 4, H-1111 Budapest, Hungary

**Keywords:** cancer, Warburg effect, mutant KRAS, PKM2, GLUT1, autophagy, chloroquine, ascorbate, systems biology

## Abstract

The Warburg effect has been considered a potential therapeutic target to fight against cancer progression. In KRAS mutant cells, PKM2 (pyruvate kinase isozyme M2) is hyper-activated, and it induces GLUT1 expression; therefore, KRAS has been closely involved in the initiation of Warburg metabolism. Although mTOR (mammalian target of rapamycin), a well-known inhibitor of autophagy-dependent survival in physiological conditions, is also activated in KRAS mutants, many recent studies have revealed that autophagy becomes hyper-active in KRAS mutant cancer cells. In the present study, a mathematical model was built containing the main elements of the regulatory network in KRAS mutant cancer cells to explore the further possible therapeutic strategies. Our dynamical analysis suggests that the downregulation of KRAS, mTOR and autophagy are crucial in anti-cancer therapy. PKM2 has been assumed to be the key switch in the stress response mechanism. We predicted that the addition of both pharmacologic ascorbate and chloroquine is able to block both KRAS and mTOR pathways: in this case, no GLUT1 expression is observed, meanwhile autophagy, essential for KRAS mutant cancer cells, is blocked. Corresponding to our system biological analysis, this combined pharmacologic ascorbate and chloroquine treatment in KRAS mutant cancers might be a therapeutic approach in anti-cancer therapies.

## 1. Introduction

Any difference in the behaviour of cancer cells compared to normal cells gives the opportunity to find them and to fight against them. This statement had already been recognised in the first decades of the twentieth century in the golden age of biochemistry. The great German biochemist, Otto Heinrich Warburg, found that cancer cells of different origin could be characterised by a glucose consumption one order of magnitude higher than normal cells. Approximately two-thirds of this high amount of glucose is converted to lactate [1,2]. Interestingly, the elevated glucose consumption was not accompanied by elevated respiration (O_2_ consumption), indicating that glucose processing occurred by lactic acid fermentation bypassing the entry of pyruvate into the TCA cycle [3]. Due to the unchanged O_2_ consumption (respiration), the phenomenon was called aerobic glycolysis. The increased production of lactic could not be avoided by higher O_2_ consumption; therefore, Warburg concluded that the respiration must have been damaged in cancer cells [4]. From the 1950s, the mitochondrial defects in cancer cells have been debated [5,6,7]; several oncogenic nuclear and mitochondrial DNA mutations in proteins involved in respiration have been described. However, in general, these mutations affect a limited range of cancer types [3].

It seems that there is no switch between oxidative phosphorylation and aerobic glycolysis, but they occur simultaneously in cancer cells [8]. Based on the in vitro results of Warburg and co-workers, the energetic background of the effect was calculated [3]. According to the calculation, under normoxic conditions, during the (same) time required for the complete oxidative metabolism of one molecule of glucose yielding 36 molecules of ATP, 10 more glucose molecules are converted via the aerobic glycolytic pathway to 20 molecules of lactate yielding an additional 20 molecules of ATP in cancer cells. While under anoxic conditions, the conversion of 13 molecules of glucose to 26 molecules of lactate yields the production of 26 molecules of ATP. On the basis of the results of Warburg and co-workers, cancer cells consume 70 mg (0.388 mmol) of glucose and release 46 mg (0.511 mmol) of lactate per 100 mL of blood [2], which means that 0.2555 mmol of glucose is metabolised via the aerobic glycolysis to lactate yielding 0.511 mmol of ATP, and 0.133 mmol of glucose was fully metabolised via the oxidative phosphorylation yielding 4.788 mmol of ATP [3]. In summary, under the same time conditions, cancer cells produced 9.64% more ATP than their normal counterparts (under normoxic conditions). An in vitro study on LN18 human glioblastoma cells reported relatively close results: a 13% increase in ATP production [9]. The coexistence of oxidative respiration and glycolysis, the so-called hybrid metabolic state, has been reported in aggressive tumour cell lines such as aggressive triple-negative breast cancer that highlights its importance in tumorigenesis [10].

However, ATP production could just be one side of the story. Continuous proliferation requires the continuous generation of biosynthetic precursors for the nucleic acid, amino acid, and lipid synthesis. The continuous aerobic glycolysis results in the accumulation of glycolytic intermediates which boost the pentose phosphate pathway, hence increasing the formation of NADPH and ribose-5-phosphate, which are precursors for the synthesis of phospholipids and nucleic acids [11]. NADPH enables cancer cells to recycle the antioxidant glutathione (GSH), which plays a crucial role in the maintenance of intracellular redox status and protects cancer cells against oxidative damage [10,12]. Furthermore, lactate produced via the aerobic glycolysis makes the tumour microenvironment acidic, which promotes tumour migration and invasion [11].

The above-mentioned hybrid metabolism enables cancer cells to switch between glycolytic and oxidative pathways that contribute adaptations to the various tumour microenvironments, such as hypoxia and acidic conditions [10]. All these observations emphasise the importance of the Warburg effect and the hybrid metabolism as a potential therapeutic target to fight against cancer progression.

Although this aerobic glycolysis is disadvantageous in general, it can be used for the detection of cancer cells with altered metabolism. The enhanced glycolysis is accompanied by the upregulation of different glucose transporters, such as GLUT1, 3 and 4. This increased uptake and utilisation of glucose by tumours gives the basis for the application of the glucose analogue 2-[^18^F]Fluoro-2-deoxyglucose in PET imaging the tumours in the body [13,14]. Necessarily, other tissues characterised by high metabolic activity are also labelled strongly [13].

The hybrid metabolic phenotype which enhances the metabolic plasticity of tumour cells and hence supports cancer invasion, metastasis, and chemoresistance is not a general feature of cancer cells. Furthermore, metabolic phenotypes in different tumours, and even in the same type of tumour, are heterogeneous [10]. Thus, the elucidation of the regulation of the Warburg effect and the examination of the inter-relationships of different regulating factors are crucial to find the weak point of tumours.

The first regulated point is the transport of glucose into the cells. GLUT1 and sodium-glucose linked transporter (SGLT1) are overexpressed in most cancer cells [10]. Furthermore, the high expression of GLUT1 and 3 is associated with elevated glucose uptake and activated oncogenic signalling pathways and growth [15,16]. The expression of two or three enzymes of the glycolytic pathway is enhanced in tumour cells. The first enzyme of the pathway is hexokinase, that has four isoforms, (HK 1–4). Among them, HK2 is expressed in insulin-sensitive adipose and muscle tissues, and it is overexpressed in several tumour cells [10]. The next—well regulated—phosphorylation step, the conversion of fructose-6-phosphate to fructose-1,6-biphosphate, is catalysed by phosphofructokinase 1 (PFK1). Its activity is regulated inter alia by the level of fructose-2,6-biphosphate, which is also produced from fructose-6-phosphate by phosphofructokinase 2 (PFK2). The reported overexpression of PFK2 in cancer cells causes the high level of fructose-2,6-bisphosphate, which activates PFK1, resulting ATP-level independent high glycolytic rates [10,17]. The final, regulated step is the conversion of phosphoenolpyruvate to pyruvate and ATP by pyruvate kinase (PK). The activity of PK is affected by the cellular pH and ATP/AMP ratio and it has four isoforms (PKL, PKR, PKM1,2). Among these, PKM2 is overexpressed in cancer cells [18]; furthermore, the PKM2 monomer can translocate into the nucleus where it behaves as a histone kinase and upregulates the expression of c-Myc and cyclin D1 [19,20]. Myc oncogene encodes the transcription factor c-Myc that is involved in the control of cellular growth and metabolism. Its expression is upregulated in many types of cancers, and its overexpression results in the enhanced expression of glucose transporter GLUT1 and elevated activity of the glycolytic enzymes (HK2, PFK, enolase1) and LDHA with the overproduction of lactic acid [21]. Furthermore, c-Myc is also linked to the increased generation of mitochondrial ROS and the upregulation of pyruvate dehydrogenase kinase1 [21].

The members of the RAS family have key roles in the modulation of cell survival, proliferation, and differentiation. Mutations which activate the members of the family can be found in as much as 20–30% of all human tumours, and mutations in the KRAS isoform can be found in 95% of pancreatic ductal adenocarcinoma and the vast majority of colon and lung tumours [22]. In KRAS mutant cells, PKM2 is hyper-activated, and it induces GLUT1 expression via the oncogenic transcription factor, c-Myc; therefore, KRAS has been closely involved in the initiation of Warburg metabolism. Although both PKM1 and PKM2 originate from the same PKM pre-mRNA as a result of alternative splicing, it seems that PKM1 has hardly any effect on cell proliferation. Furthermore, the replacement of PKM2 with PKM1 results in the inhibition of glycolysis in lung cancer cells and suppresses tumour xenograft formation in nude mice [10,23]. PKM2 clearly promotes the Warburg effect and cell cycle progression, thus it is an ideal target in anti-tumour therapies. Recently, it has been shown that pharmacologic ascorbate impairs the Warburg effect in KRAS mutant cells and xenografts by resulting in the downregulation of both GLUT1- and PKM2-dependent protein expression, suggesting its positive role in cancer therapies [24].

Interestingly, the key controller of cellular metabolism, called mTOR (mammalian target of rapamycin), is also activated in KRAS mutants [25]. It has also been shown that the Akt/mTOR pathway was inhibited in cases of PKM2 knockdown, supposing that PKM2 has a positive effect on the mTOR pathway [26,27]. In addition, the mTOR pathway also enhances aerobic glycolysis by upregulating PKM2 via c-Myc induction [28], suggesting that inhibition of the mTOR pathway by pharmacological components (such as RapaLinks [29]) might have therapeutic importance in cancer treatment. These results also suggest that PKM2 might have a central role in controlling GLUT1 transport in KRAS mutant cells. Although mTOR is a well-known inhibitor of autophagy-dependent survival in physiological conditions, many recent studies have revealed that autophagy becomes hyper-active in KRAS mutant cancer cells [30]. Although the role of autophagy seems to be tumour suppressive in the initial stages of cancer, the hyper-active autophagy-dependent self-cannibalism has serious negative effects in tumour progression in the later stages. Therefore, the downregulation of autophagy by using various drugs (i.e., chloroquine or hydroxychloroquine) might have important roles in cellular survival [31].

In the present study, a systematic analysis was performed to explore even further the possible therapeutic strategies in KRAS mutant cancer cells. A mathematical model of a minimal network was developed, and computer simulations were carried out, which suggest that the positive feedback loops of both KRAS and mTOR pathways have crucial roles in determining the GLUT1 transport and autophagy induction upon cancer development. Our dynamical analysis suggests that the downregulation of KRAS, mTOR and autophagy are crucial in anti-cancer therapy. This tiny model makes it possible to test the phenotypes of various drug treatments such as the addition of pharmacologic ascorbate and chloroquine. Moreover, the positive role of novel combinations of these above-mentioned drugs in cancer development is also introduced in KRAS mutant cells, suggesting the importance of our theoretical analysis.

Mathematical modelling of Warburg metabolism has already been addressed in several studies, mostly for the purpose of a deeper understanding of tumour metabolism [32,33,34,35] or pathway engineering [36]. However, they mostly focus on specific participants in the metabolic pathways rather than the regulatory network of metabolism. To the best of our knowledge, a dynamical analysis modelling a regulatory network for the Warburg effect in KRAS mutant cells has not yet been performed for anti-tumour therapeutic purposes, further reinforcing the novelty of our study.

## 2. Materials and Methods

### 2.1. Mathematical Modelling

To study the dynamical characteristic of the control network, it was converted to a set of nonlinear ordinary differential equations (ODEs) and analysed using the techniques of dynamical system theory [37,38,39]. It has been demonstrated several times that a simple model such as ours can provide a proper description when studying cellular control networks [40,41,42]. For details about equations, codes and software, see Section 2.2. Descriptions of the theoretical analysis are in Appendix A and Appendix B. Dynamical simulations were achieved by the usage of the freely available program XPP-AUT, 8.0 (Department of Mathematics, Pittsburg University, Pennsylvania, USA) [37]. 

### 2.2. Describing the Theoretical Analysis

Here, we briefly describe the mathematical approach used to study the autophagy induction. A system-level view can be developed by bringing together the components and interactions reported in the literature. Such a network can be translated into a set of mathematical equations that describe how each component concentration/activity in the network changes with the time. The rate of change of a component is described by an ordinary differential equation (ODE) based on biochemical reaction kinetics. Each biochemical reaction is represented as a term on the right-hand side of the ODE for a component participating in the reaction [38,43]. Each reaction in the network can be described either by using laws of mass action or Michaelis–Menten kinetics [37,44,45]. The steps of modelling are as follows: (1) collect relevant experimental data on the processes of system to be investigated; (2) characterise the system components with ordinary differential equations, containing various parameters; (3) solve the system of differential equations in a numerical manner, that can be performed by computer using an estimated parameter set; (4) vary the parameters, to reach an agreement between calculated and experimental results; (5) make predictions using the validated mathematical model.

Our differential equation describing the temporal activity changes of the given component is composed of two parts: activation and inactivation terms (differential equations used for the simulations can be found in the Appendix A). Usually, protein activity can be described either by mass action or Michaelis–Menten kinetics [37,46]. For example, if the activity of protein is controlled by covalent modification involving multi-site phosphorylation, Michaelis–Menten kinetics provide a good approximation for the process [47,48]. The value of parameters (rate constants, Michaelis constants) and initial conditions have to be specified in order to solve ODEs. The non-linear nature of biological processes makes it difficult to find the solution of ODEs analytically; hence, the equations have to be solved numerically. The equations can be solved using different numerical integration methods that are implemented as solvers in many of the freely available computer software programs.

Solving a set of non-linear ODEs gives the time evolution of the protein concentration/activity called time courses. Furthermore, ODEs can be solved to obtain the input–output relationship called the signal–response curves or as one parameter bifurcation diagram [37,38,39]. An input is the signal strength that is varied to obtain the steady state behaviour of the control system. This helps to capture the qualitative changes in the behaviour of the system. For example, the system behaviour can become abrupt and discontinuous when signal strength is increased from a low value to high value. A point at which such a qualitative change in the system occurs is defined as the bifurcation point [38].

In this study, the temporal profiles and signal–response curves were computed numerically using XPP-AUT, 8.0 (Department of Mathematics, Pittsburg University, Pennsylvania, USA). All the simulations presented in the text are based on XPP codes found in the Appendix B. The rate constants (k) have the dimension of relative (time unit)^−^^1^ and Michaelis constants (J) are dimensionless. The protein activities are given in arbitrary units (a.u). The starting parameter set was able to refer to physiological conditions. Physiological conditions refer to those conditions which occur in nature for the cellular system and no inner or outer stress is induced. The parameters values were perturbed to capture all the possible qualitative behaviours that the given network can exhibit (e.g., with the addition of ascorbate, the inactivation rate of KRAS was increased; in cases of chloroquine treatment, the inactivation rate of mTOR was induced) and the level of autophagy was decreased [49,50].

## 3. Results

### 3.1. KRAS Mutant Cancer Cells Exhibit High Levels of Both GLUT1 and Autophagy

In KRAS mutant cells, PKM2 is hyper-activated, and it induces GLUT1 expression via promoting by transcription the oncogenic transcription factor, c-Myc [26,51]. Moreover, c-Myc is able to upregulate PKM2, generating a positive feedback loop in the control network [22]. PKM2 also has a positive effect on its own activation by inducing the phosphorylation of ERK1/2 in the KRAS pathway [51]. Recently, it has been shown that the addition of Vitamin C in pharmacologic concentrations selectively kills KRAS mutant cells, resulting in the downregulation of both GLUT1- and PKM2-dependent protein expression, suggesting its positive role in anti-tumour therapies [22,24].

Interestingly, in KRAS mutant cells, mTOR becomes up-regulated via PI3K [25]; meanwhile, autophagy also becomes hyper-activated [52,53,54]. Moreover, PKM2 knockdown inhibits the Akt/mTOR pathway, suggesting that PKM2 has a positive effect on the mTOR pathway [26,27]. Not only does PKM2 promote mTOR, but the mTOR pathway also enhances aerobic glycolysis by upregulating PKM2 via c-Myc induction, generating another positive feedback loop in the control network [28]. These data also suggest that mTOR inhibitors combined with the downregulation of autophagy might be a potential therapeutic option for cancer treatment because of the strong autophagy dependence of KRAS mutant cancer cells.

**Figure 1:**
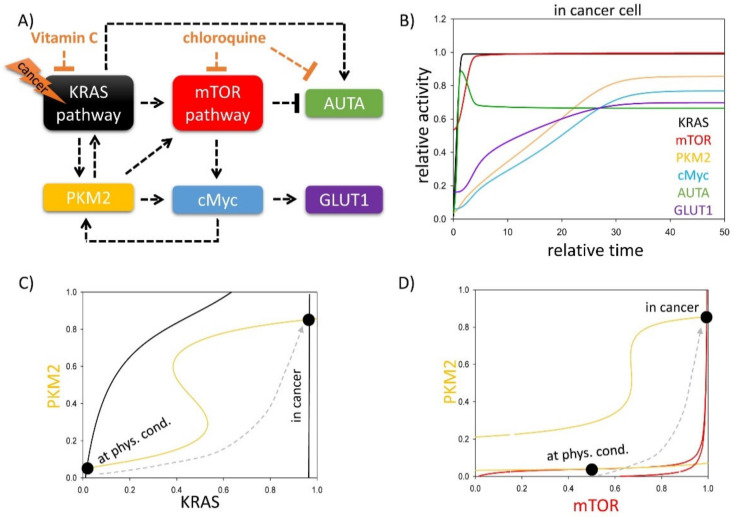
Both KRAS and mTOR pathways have key roles in determining autophagy induction and GLUT1 transport upon various cellular stress events. (**A**) The key regulatory components and their interactions at various stress events. Autophagy refers to those elements which can induce autophagy with respect to cellular stress, while cell death inducer means the sum of the activators of cellular cell death process. Dashed lines show how the molecules can influence each other. Blocked end lines denote inhibition. (**B**) The computational simulations are determined in KRAS mutant cancer cells. The temporal dynamics are simulated with karas = 1. The relative activity of KRAS, PKM2, mTOR, cMyc, autophagy (AUTA) and GLUT1 is shown. The (**C**) PKM2–KRAS and (**D**) PKM2–mTOR signal–response curves are plotted in physiological conditions (karas = 0.1) and in cancer cells (karas = 1). Intersections of nullclines represent the stable (filled circle) steady states. Trajectories are depicted with dashed grey lines. For detailed descriptions of the elements and constants of the theoretical models, see Table A1 and Table A2, respectively.

To provide a detailed qualitative description about the control network and to understand the dynamic characteristics of GLUT1 transport in KRAS mutant cancer cells, a simple mathematical model was built (for details, see Materials and Methods), containing only the main regulatory elements of the regulatory network (Figure 1A). In this simple model, we supposed that both KRAS and mTOR pathways have crucial roles in determining the autophagy induction and GLUT1 transport upon various cellular stress events. In our wiring diagram, four overlapping positive feedback loops were operating: (1) PKM2→KRAS→PKM2; (2) KRAS→mTOR→c-Myc→PKM2→KRAS; (3) mTOR→c-Myc→PKM2→mTOR; (4) PKM2→c-Myc→PKM2.

To reveal the exact role of the regulatory elements in the stress response mechanism, first, signal–response curves were generated (Figure 1C,D). Assuming that the other components were in steady state, the dynamical features of the non-linear ordinary differential system were analysed in coordinated systems spanned by KRAS and PKM2 (Figure 1C) or mTOR and PKM2 (Figure 1D). Along the so-called balance curve, the rate of the active form is exactly balanced by its rate of inactive form of the given component. The intersections between two balance curves are called equilibrium points: here, the system has steady state solutions, which can represent the observable physiological states of the regulatory system. The over-lapping positive feedback loops generate bistability in the control network. In physiological conditions, the system has one stable steady state with low levels of active PKM2 and KRAS; meanwhile, a high level of mTOR is observed which blocks autophagy (see the black dots called “at. phys. cond.” on Figure 1C,D). In the case of KRAS mutation due to the S-shaped form of the PKM2 nullcline, the cells quickly generate a switch-like activation of another steady state with high levels of PKM2, KRAS and mTOR (see the black dots called “in cancer” on Figure 1C,D) upon tumour generation. In this case, the hyper-activation of PKM2 helps the drastic up-regulation of GLUT1 via c-Myc. Although mTOR becomes hyper-activated and tries to block autophagy, KRAS is able to directly turn on the self-digestive process [52], resulting in an interesting phenotype, when both mTOR and autophagy are active (Figure 1B).

### 3.2. PKM2 Level Directly and Indirectly Affects Stress–Response Mechanisms

**Figure 2:**
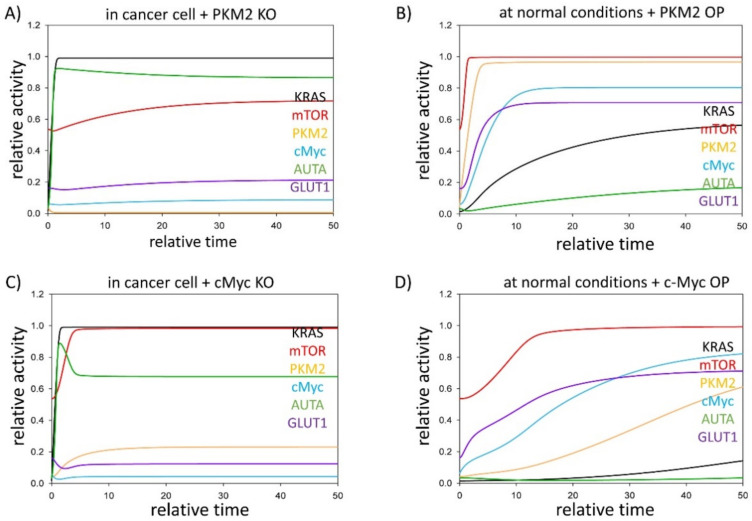
PKM2 level might have a therapeutic role in KRAS mutant cancer cells. The computational simulations were determined in normal conditions when PKM2 is (**A**) depleted (PKM2T = 0.01) in cancer cells (karas = 1), or (**B**) over-expressed (kapkm = 0.5) in physiological conditions (karas = 0.1). The dynamical characteristic of c-Myc is plotted when tumour progression is combined with cMyc: (**C**) depletion (cMycT = 0.1, karas = 1) or (**D**) c-Myc is over-expressed in physiological conditions (cMycT = 10, karas = 0.1). The relative activity of KRAS, PKM2, mTOR, cMyc, autophagy (AUTA) and GLUT1 is shown. For detailed description of the elements and constants of the theoretical models, see Table A1 and Table A2, respectively.

PKM2 was present in all four positive feedback loops of our simple model; therefore, we assumed that PKM2 might be the key switch of the control network. We suggest that PKM2 has essential roles, not only directly controlling mTOR and KRAS pathways, but indirectly via c-Myc up-regulation in the stress response mechanism. To further explore the effect of PKM2 in KRAS mutant cancer cells, time course simulations were carried out where either PKM2 or c-Myc were systematically downregulated in cancer, while they were separately up-regulated in physiological conditions (Figure 2).

Corresponding to the experimental data [26,51], the relative activity of both PKM2 and c-Myc are high in tumour cells, resulting in intensive GLUT1 expression (Figure 1B); in contrast, in the absence of either PKM2 or c-Myc, the GLUT1 level remains low [26,55]. Here, we also confirm with theoretical analysis that although either PKM2 or c-Myc are knocked down, the KRAS pathway is still active and has a positive effect on the mTOR pathway. Moreover, KRAS also keeps autophagy active (Figure 2A,C) [52,53,54].

In cases when either PKM2 or c-Myc are over-expressed in physiological conditions, the cells show similar properties to cancer cells (Figure 2B,D). In the case of c-Myc overexpression, the KRAS pathway remains inactive, but c-Myc alone is sufficient to hyper-activate the response mechanism. PKM2, c-Myc and mTOR are all highly expressed; therefore, GLUT1 expression is significantly up-regulated, while the mTOR pathway keeps autophagy inactive.

Our data confirm that PKM2 and its direct substrate, c-Myc, are major players of KRAS mutation-induced cancer by performing various metabolic (i.e., positive effect on GLUT1) and non-metabolic (i.e., up-regulation of autophagy) functions. These results also suggest that PKM2 or c-Myc might provide a new horizon in anti-tumour therapies.

### 3.3. Either KRAS- or mTOR Pathway Inhibition Downregulates GLUT1 Level in Cancer Cells

**Figure 3:**
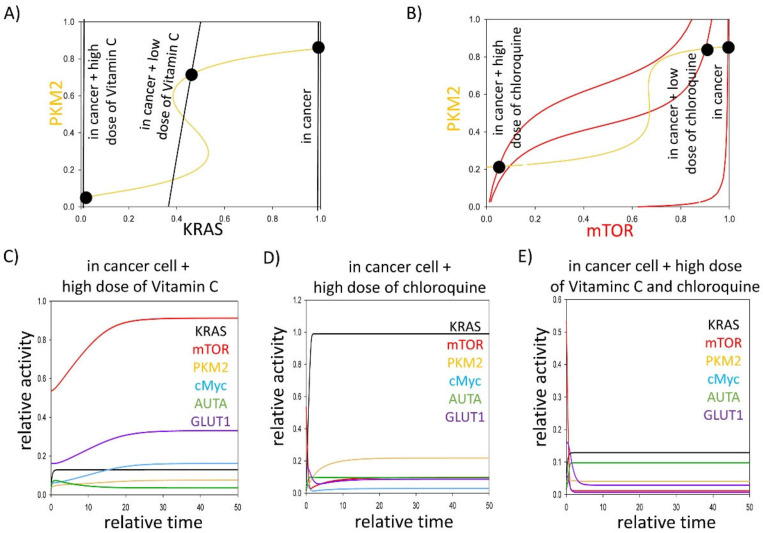
Affecting either KRAS or mTOR pathways might have therapeutic roles in treating cancer cells. The (**A**) PKM2–KRAS and (**B**) PKM2–mTOR signal–response curves are plotted in cancer cells (karas = 1) combined with addition of various doses of (**A**) pharmacologic ascorbate (kiras’ = 1, kiras’ = 1.5) or (**B**) chloroquine (kimr’ = 1, kimr’ = 1.5). Intersections of nullclines represent the stable (filled circle) steady states. The computational simulations are determined in cancer cells (karas = 1) combined with the addition of (**C**) pharmacologic ascorbate (kiras’ = 1.5), or (**D**) during chloroquine treatment (kimr’ = 1.5, AUTAT = 0.1), or (**E**) in a combined treatment of pharmacologic ascorbate and chloroquine (kiras’ = 1.5, kimr’ = 1.5, AUTAT = 0.1). The temporal dynamics are simulated with karas = 1. The relative activity of KRAS, PKM2, mTOR, cMyc, autophagy (AUTA) and GLUT1 is shown. For detailed description of the elements and constants of the theoretical models, see Table A1 and Table A2, respectively.

Questions immediately arise as to what types of various anti-tumour therapies can be used in KRAS mutant cancer cells. Recently, it has been shown that pharmacologic ascorbate impairs the Warburg effect in KRAS mutant cells and xenografts by resulting in the downregulation of both GLUT1- and PKM2-dependent protein expression, suggesting its positive role in cancer therapies [24]. Moreover, it is well known that mTOR is upregulated in KRAS mutant cancers, but KRAS can directly turn on the self-digestive process, keeping the autophagy active. Due to the strong autophagy dependence of KRAS mutant tumours, their inhibitors, such as chloroquine, have been used for treating cancer in the clinic [56,57]. To understand the dynamical characteristics of stress–response mechanisms in the addition of either ascorbate (restoring glycolytic flux) or chloroquine (downregulating both mTOR and autophagy), signal–response curves were generated (Figure 3A,B).

In cases of cancer, both mTOR and KRAS intersect the PKM2 nullcline in a stable state, where each of the three components has high activities. Either the addition of pharmacologic ascorbate or chloroquine can impair KRAS mutation in a dose-dependent manner (see black dots called “in cancer + low dose of ascorbate/chloroquine” and “in cancer + high dose of ascorbate/chloroquine” on Figure 3A,B). Namely, the stable state moves to the left on the signal–response curves. Reaching a proper level of these chemical compounds, the switch-like inactivation of PKM2 can be observed. At the same time, mTOR and KRAS activity are both switched off, forming a new stable state with low activity of mTOR, KRAS and PKM2.

Our computer simulations have revealed that PKM2 downregulation induced by the high level of ascorbate or chloroquine resulted in the inhibition of c-Myc and GLUT1 expression (Figure 3C,D); however, the addition of pharmacologic ascorbate is not able to block mTOR pathway. In contrast, chloroquine directly inhibits both mTOR and autophagy, however the KRAS pathway remains active (Figure 3D). To further understand their roles in the stress–response mechanism, we predicted that a combined treatment of pharmacologic ascorbate and chloroquine are able to block mTOR and KRAS pathways, which results in the downregulation of both GLUT1 and autophagy (Figure 3E). Downregulation of autophagy can be an effective anti-cancer approach because of the strong autophagy dependence of KRAS mutant cancer cells [52,58].

Corresponding to our biological system analysis, we propose that a combined addition of pharmacologic ascorbate and chloroquine in KRAS mutant cancer cells, with the inhibition of GLUT1 and downregulation of autophagy, might be a therapeutic approach in anti-cancer therapies.

## 4. Discussion

RAS is the most frequently mutated gene in human cancer. Mutations which activate members of the RAS family (KRAS, HRAS, and NRAS) can be found in as much as 20–30% of all human tumours [22]. The members of the family have key roles in the modulation of cell survival, proliferation, and differentiation via molecular pathways such as PI3K–AKT–mTOR. Although there are high differences in the mutated isoforms (HRAS, NRAS or KRAS) and mutational frequency across different tumour types, mutations in the KRAS isoform can be found in 95% of pancreatic ductal adenocarcinoma and the vast majority of colon and lung tumours, while the rates of oncogenic mutation occurring in the NRAS and HRAS isoforms are much lower [22]. The mutant KRAS has been closely involved in the initiation of Warburg metabolism, the key role of KRAS signalling in the homeostasis of aerobic glycolysis which has been described in different types of cancer [22]. In KRAS mutant cells, PKM2 is hyper-activated, and it induces GLUT1 expression via the oncogenic transcription factor, c-Myc [26] and c-Myc is able to upregulate PKM2, generating a positive feedback loop [22]; furthermore, PKM2 has a positive effect both on the mTOR pathway and also on its own activation by inducing the phosphorylation of ERK1/2 [26,55]. A simple mathematical model was built, containing the main regulatory elements of the regulatory network to provide a detailed qualitative description about the control network in KRAS mutant cancer cells. The simplified regulatory network contains KRAS, GLUT1, c-Myc, PKM2 and mTOR. Although mTOR is a well-known inhibitor of autophagy-dependent survival in physiological conditions, many recent studies have revealed that autophagy becomes hyper-active in KRAS mutant cancer cells [30]. Although the role of autophagy seems to be tumour-suppressive in the initial stages of cancer, the hyper-active autophagy-dependent self-cannibalism has serious negative effects in tumour progression in the later stages. Therefore, the downregulation of autophagy by using various drugs (i.e., chloroquine or hydroxychloroquine) might have an important role in cellular survival [31].

It was found that the system has one stable steady state in physiological conditions with low levels of active PKM2 and KRAS; meanwhile, a high level of mTOR is observed which blocks autophagy (Figure 1C,D). However, the cells quickly generate a switch-like activation of another steady state with high levels of PKM2, KRAS and mTOR in the case of KRAS mutation (Figure 1B). PKM2 via c-Myc helps the drastic upregulation of GLUT1, while autophagy becomes activated via KRAS-dependent activation, despite the fact the mTOR is also hyper-activated. Our analysis suggests an abnormal phenotype when KRAS and mTOR pathways and autophagy-dependent self-cannibalism are also hyper-activated, resulting in a high GLUT1 level.

Our findings are in concordance with experimental observations (see Table 1); in a murine pancreatic ductal adenocarcinoma model, the mutated KRAS maintained tumour growth by the stimulation of glucose uptake and channelled glucose intermediates into the hexosamine biosynthesis pathway and pentose phosphate pathway to provide further biosynthetic precursors [59]. Similarly, in KRAS mutant colorectal cancer cells, the upregulation of GLUT1 and consequent fluorodeoxyglucose accumulation was observed by PET [60,61]. Furthermore, GLUT1 was among the three genes which were consistently upregulated in cells with KRAS or BRAF mutations. The elevated glucose uptake in mutant cells resulted in cell survival in low-glucose conditions, while only a few cells survived the low-glucose conditions with wild-type KRAS. Additionally, survivors acquired KRAS mutations not present in their parents, suggesting that glucose deprivation can drive the acquisition of (KRAS) pathway mutations in human cancer cells [62]. Furthermore, the basal level of autophagy was found to be elevated in several PDAC cell lines and primary tumours [58].

The key role of PKM2 in the (regulation of) Warburg effect was not accidentally pointed out [20,22,26]. PKM2 was present in all the four positive feedback loops of our model; thus, it was rightly assumed that PKM2 can be the key switch of the control network. Furthermore, PKM2 may also have an essential indirect role via c-Myc upregulation in the stress–response mechanism. This potential central role of PKM2 in KRAS mutant cancer cells was studied through time course simulations where either PKM2 or c-Myc were systematically downregulated in cancer, while they were separately upregulated in physiological conditions. Our results reinforce the experimental data of Dey et al. [26], because the relative activities of both PKM2 and c-Myc are high in tumour cells, resulting in intensive GLUT1 expression, while in the absence of either PKM2 or c-Myc,s GLUT1 levels remain low (Figure 2A,C). This is easily understandable, because PKM2 in the nucleus is a coactivator of β-catenin and induces the expression of c-Myc [19] that results in the upregulation of GLUT1 [20]. Although the knock-down of PKM2 and c-Myc results in the downregulation of GLUT1, the KRAS pathway still remains active and has a positive effect on the mTOR pathway; meanwhile, KRAS is able to induce autophagy (Figure 2A,C). At this point, the advantages of our simulation can be mentioned: because PKM2 and PKM1 originate from the same *PKM* pre-mRNA [10], it is challenging to produce PKM2 knockout mutants. Necessarily, there are results on PKM2 silenced cell lines, however a low amount of PKM2 can be found in these lines. Interestingly, the silencing of PKM2 or c-Myc resulted in the significant downregulation of GLUT1 which is in concordance with our results; however, the silencing was accompanied by an increase in autophagic cell death [26,55]. The regulation of autophagy might be also explained by the leaky PKM2 expression.

The real advantage of our in silico approach is shown in the next step, in which PKM2 or c-Myc are over-expressed in physiological conditions. In either case, the cells show similar properties to cancer cells, although a slight difference can be observed between the overexpression of PKM2 and c-Myc. When PKM2 is overexpressed, mTOR and KRAS pathways are quickly activated due to the overlapping positive feedback loops of the control network which generate a remarkable toggle switch. In the case of c-Myc overexpression, mTOR is activated similarly to the overexpression of PKM2, however the activity of KRAS lag behind the cells overexpressing PKM2 (Figure 2B,D). The highly expressed PKM2, c-Myc and mTOR are sufficient even in the absence of KRAS to keep the GLUT1 upregulated, while the autophagy remains inactive. Our data confirm that PKM2 and its direct substrate, c-Myc, are major players of KRAS mutation induced cancer by performing various metabolic (i.e., positive effect on GLUT1) and non-metabolic (i.e., up-regulation of autophagy) functions. These results also suggest that PKM2 or c-Myc might provide a new horizon in anti-tumour therapies. The observation that the knockdown or the pharmacologic inhibition of PKM2 by shikonin in hepatocellular carcinoma cell lines resulted in a less proliferative and aggressive phenotype as well as improved drug sensitivity to both doxorubicin and cisplatin [64] emphasises the relevance of PKM2 as a therapeutic target.

What type of anti-tumour therapies are worth using in KRAS mutant cancer cells? Both mTOR and autophagy are upregulated in KRAS mutant cancer cells; therefore, chloroquine has been used for treating cancer in the clinic [56,57]. Furthermore, it has recently been shown that pharmacologic ascorbate kills colorectal cancer cells depending on the KRAS mutational status [64], and it impairs the Warburg effect by resulting in the downregulation of both GLUT1- and PKM2-dependent protein expression [24]. Our analysis suggests that a high dose of ascorbate seems to be important in cancer treatment (Figure 3A). These results also confirm that finding the proper concentration of Vitamin C in anti-tumour therapies is pivotal and requires experimental confirmation. To gain further insights into the dynamic characteristics of stress–response mechanisms with the addition of either ascorbate (restoring glycolytic flux) or chloroquine (downregulating both mTOR pathway and autophagy), signal–response curves were generated (Figure 3A,C).

Here, we suggested that either the addition of ascorbate or chloroquine can impair KRAS mutation in a dose-dependent manner (Figure 3A,B). Reaching a proper level of these chemical compounds, the switch-like inactivation of PKM2 can be observed. Our computer simulations have revealed that PKM2 downregulation induced by a high level of ascorbate or chloroquine resulted in the inhibition of c-Myc and GLUT1 expression (Figure 3C,D). In concordance with our results, the pharmacologic ascorbate treatment of KRAS mutant colorectal cancer cells caused the dramatic downregulation of GLUT1 [24]. The downregulation of GLUT1 by pharmacologic ascorbate occurred at both mRNA and protein level. The impressive inhibition of GLUT1 expression could also be observed in pharmacologic ascorbate-treated murine KRAS mutant SW480 xenografts [24]. The pharmacologic ascorbate treatment did not cause any change in the mRNA expression of PKM2, but it inhibited the phosphorylation of PKM2 at Ser37 [24] that halted the nuclear translocation of PKM2, suggesting that pharmacological ascorbate exerted its selective anti-tumour activity at least partly through the Warburg effect.

Interestingly, the addition of pharmacologic ascorbate is not able to block the mTOR pathway (Figure 3C). In contrast, chloroquine directly inhibits mTOR and autophagy, however the KRAS pathway remains active (Figure 3D). Subsequently, we predicted that a combined treatment of pharmacologic ascorbate and chloroquine are able to block both KRAS and mTOR pathways (Figure 3E). In this case, no GLUT1 expression is observed; furthermore, autophagy is also inhibited that can result in a real win situation because of the strong autophagy dependence of KRAS mutant tumours [52,58].

Corresponding to our biological system analysis, we propose the combined addition of pharmacologic ascorbate and chloroquine in KRAS mutant cancer treatment. By the inhibition of GLUT1 and the downregulation of autophagy, this might be a therapeutic approach in anti-cancer therapies.

## Figures and Tables

**Table 1:** Experimental observations underlying our model.

Model Organism (Mutant)	Treatment	Observed Phenomenon (after Treatment)	Reference
Xenografts (KRAS mutant G12D)		The mutated KRAS maintains tumour growth by the stimulation of glucose uptake and channels glucose intermediates into the hexosamine biosynthesis pathway and PPP to provide further biosynthetic precursors	[59]
HCT116 and DLD-1 (KRAS mutants); clinical CRC samples		CRC cells with mutated KRAS increased 18F-FDG accumulation by upregulating GLUT1	[60]
HCT116 and DLD-1 (KRAS mutants); xenografts		Glucose deprivation can drive the acquisition of KRAS pathway mutations in human cancer cells	[62]
U251 human glioblastoma multiforme cells (ERK2, MEK1 mutants), U87/EGFR cells (PKM2 mutants)	EGF stimulation	Nuclear translocation of PKM2 is a cause of the Warburg effect; nuclear PKM2 induces c-Myc expression, resulting in the upregulation of GLUT1, LDHA, and, in a positive feedback loop, PTB-dependent PKM2 expression	[51]
U87/EGFR GBM cells, U251 GBM cells, and human embryonic kidney 293T	EGF treatment	Monomeric PKM2 translocates into the nucleus, where it functions as a histone kinase and upregulates the expression of c-Myc and cyclin D1	[20]
U87/EGFR cells	EGF treatment	PKM2 upregulates the expression of c-Myc and cyclin D1	[19]
AGS and HGC-27 gastric cancer cells	PKM2 and c-Myc lentivirus	PKM2 and c-Myc were upregulated in human gastric cancer; knockdown of c-Myc in gastric cancer cells suppressed cell proliferation capacity and glycolysis level, the inhibitory effects on gastric cancer cells upon co-knockdown of PKM2 and c-Myc were more obvious compared with knockout of PKM2 or c-Myc alone	[55]
HCT116 and DLD1 (KRAS mutants) xenografts derived from parental HCT116 and VACO432 cell lines	High dose Vitamin C	Vitamin C selectively kills KRAS and BRAF mutant colorectal cancer cells	[63]
SW480 (KRAS mutation (G12V); LoVo cancer cells (KRAS mutant G13D); xenograft (SW480)	Pharmacologic ascorbate	c-Myc is able to upregulate PKM2; in KRAS mutants, Vitamin C induces strong downregulation of the glucose transporter (GLUT1) and PKM2-PTB-dependent protein expression	[24]
NCI-H460, A549, HCT116 (KRAS mutants)		mTOR (mammalian target of rapamycin) is also activated in KRAS mutant	[25]
DU145 prostate cancer cells		Prostate cancer cells show increased proliferation because of PKM2 overexpression; PKM2 knockdown downregulated β-catenin and c-MYC expression, which in turn decreased the expression of glycolytic enzymes LDHA, GLUT1, and of HIF1α; PKM2 knockdown inhibits the Akt/mTOR pathway	[26]
HEK293T and HeLa cells		Overexpression of PKM2 activates mTORC1 signalling	[27]
MEFs, PANC-1, PC3, and HepG2		mTOR pathway also enhances aerobic glycolysis by upregulating PKM2 via c-Myc induction	[28]
T24, H1299, H460, PC-3, PANC-1, HCT116	Chloroquine	Expression of an H-rasV12 or K-rasV12 oncogene up-regulates basal autophagy, which is required for tumour cell survival in starvation and in tumorigenesis; high level of autophagy in Ras expressing cells occurred despite active mTOR, which suppresses autophagy and is activated by Ras, which suggests that the high basal autophagy caused by Ras must result from an mTOR-independent mechanism	[52]
PDAC cell lines (8988T, Panc1, pl45, HupT3, 8902, BXPC3, 10.05, 2.03); xenografts; primary tumours	Chloroquine	The basal level of autophagy was found to be elevated in several PDAC cell lines and primary tumours; PDAC cell lines exhibit a marked sensitivity to chloroquine; chloroquine treatment leads to robust tumour regression and prolonged survival in pancreatic cancer xenografts	[58]
Pa01C, Pa02C, Pa04C, Pa14C and Pa16C; PANC-1; HPAC; HPAF-II; MIA PaCa-2; SW1990; CFPAC-1; BxPC3; HEK-293T; RIE-1 cell lines; xenografts	KRAS; MEK; ERK1/2 inhibitors; hydroxychloroquine	Genetic or pharmacologic inhibition of specific autophagy regulators synergistically enhanced the ability of ERK inhibitors to mediate anti-tumour activity in KRAS-driven PDAC	[56]
Mia-PaCa2; BxPC3; PDX220; xenografts	chloroquine/ hydroxychloroquine; ARS-853 (inhibitor of KRASG12C), trametinib or cobimetinib (MEK1/2 inhibitors), or SCH772984 (ERK1/2 inhibitor)	Trametinib and chloroquine are synergistically cytotoxic to PDA cell lines in vitro and promote regression of RAS→RAF→MEK→ERK driven cancers	[57]

## Data Availability

Not Applicable.

## Abbreviations

Akt	protein kinase B
AMP	adenosine monophosphate
ATP	adenosine triphosphate
ERK1/2	extracellular signal-regulated kinases
GLUT1–4	glucose transporter isozymes 1–4
GSH	glutathione
HK 1–4	hexokinase isozymes 1–4
LDHA	lactate dehydrogenase A
mTOR	mammalian target of rapamycin
NADPH	nicotinamide adenine dinucleotide phosphate
ODE	ordinary differential equations
PET	positron emission tomography
PFK1/2	phosphofructokinase isozymes 1/2
PI3K	phosphoinositide 3-kinase
PK	pyruvate kinase
PKM1/2	pyruvate kinase isozymes M1/M2
SGLT1	sodium-glucose linked transporter
TCA cycle	tricarboxylic acid cycle

## Appendix A

Differential equations used for simulations:

(A1) $$\frac{dKRAS}{dt}=\frac{\left(karas+karas’\times PKM2\right)\times \left(KRAST-KRAS\right)}{Jkras+KRAST-KRAS}-\frac{\left(kiras+kiras’\times VitC\right)\times KRAS)}{Jkras+KRAS}$$

(A2) $$\frac{dmTOR}{dt}=\frac{\left(kamr+kamr’\times KRAS+kamr”\times PKM2\right)\times \left(mTORT-mTOR\right)}{\left(Jmr+mTORT-mTOR\right)}-\frac{kimr+kimr’\times CHL)\times mTOR}{Jmr+mTOR}$$

(A3) $$\frac{dPKM2}{dt}=\frac{\left(kapkm+kapkm’\times KRAS+kapkm”\times cMyc\right)\times \left(PKM2T-PKM2\right)}{Jpkm+PKM2T-PKM2}-\frac{kipkm\times PKM2}{Jpkm+PKM2}$$

(A4) $$\frac{dcMyc}{dt}=\frac{\left(kamyc+kamyc’\times PKM2+kamyc”\times mTOR\right)\times \left(cMycT-cMyc\right)}{Jmyc+cMycT-cMyc}-\frac{kimyc\times cMyc}{Jmyc+cMyc}$$

(A5) $$\frac{dAUTA\;}{dt}=\frac{\left(kaau+kaau‘\times KRAS\right)\times \left(AUTAT-AUTA\right)}{Jau+AUTAT-AUTA}-\frac{\left(kiau+kiau’\times mTOR\right)\times AUTA}{Jau+AUTA}$$

(A6) $$\frac{dGLUT1\;}{dt}=\frac{\left(kaglu+kaglu’\times cMyc\right)\times \left(1-GLUT1\right)}{Jglu+1-GLUT1}-\frac{kiglu\times GLUT1}{Jglu+1-GLUT1}$$

**Table A1:** The detailed description of the elements of the theoretical models and their value in physiological conditions.

Elements of the Theoretical Models	Description	Parameter Value
KRAS	The active form of KRAS	variable
KRAST	Total level of KRAS	1
PKM2	The active form of pyruvate kinase 2	variable
PKM2T	Total level of pyruvate kinase 2	1
mTOR	The active form of mTOR	variable
mTORT	Total level of mTOR	1
cMyc	The active form of cMyc	variable
cMycT	Total level of cMyc	1
AUTA	The active autophagy activator complex	variable
AUTAT	Total amount of autophagy	1
GLUT1	The active form of GLUT1	variable
VitC	Vitamin C	1
CHL	Chloroquine	1

**Table A2:** The detailed description of the constants of the theoretical models and their value in physiological conditions.

Constants of the Theoretical Models	Description	Parameter Value
karas	Background activation of KRAS	0.01; 1
karas’	PKM2-dependent activation of KRAS	0.1
kiras	Background inactivation of KRAS	0.1
kiras’	Vitamin C-dependent inactivation of KRAS	0; 1.5
Jkras	Michaelis constant of KRAS	0.1
kamr	Background activation of mTOR	0.01
kamr’	KRAS-dependent activation of mTOR	0.1
kamr”	PKM2-dependent activation of mTOR	2.25
kimr	Background inactivation of mTOR	0.1; 0.2
kimr’	Chloroquine-dependent inactivation of mTOR	0; 1; 1.5
Jmr	Michaelis constant of mTOR	0.1
kapkm	Background activation of PKM2	0.05
kapkm’	KRAS-dependent activation of PKM2	0.1
kapkm”	cMyc-dependent activation of PKM2	0.2
kipkm	Background activation of autophagy	0.2
Jpkm	Michaelis constant of PKM2	0.1
kamyc	Background activation of cMyc	0.01
kamyc’	PKM2-dependent activation of cMyc	0.2
kamyc”	mTOR-dependent inactivation of cMyc	0.2
kimyc	Background inactivation of cMyc	0.3
Jmyc	Michaelis constant of cMyc	0.1
kaau	Background activation of autophagy	1
kaau’	KRAS-dependent activation of autophagy	7.5
kiau	Background inactivation of autophagy	0.01
kiau’	mTOR-dependent inactivation of autophagy	7.5
Jau	Michaelis constant of autophagy	0.1
kaglu	Background activation of GLUT1	0.01
kaglu’	cMyc-dependent activation of GLUT1	3
kiglu	Background inactivation of GLUT1	1
Jglu	Michaelis constant of GLUT1	0.01

## Appendix B

The codes for simulating signal–response curves:

#1 CODE
# a model to generate PKM2–KRAS signal–response curves
# initial conditions
init KRAS=0, PKM2=0
# differential equations
# KRAS represents the active form of KRAS
KRAS’= (karas + karas’*PKM2)*(KRAST-KRAS)/(Jkras + KRAST-KRAS)−(kiras + kiras’*VitC)*KRAS/(Jkras + KRAS)
# PKM2 represents the active form of PKM2
PKM2’ = (kapkm + kapkm’*KRAS + kapkm”*cMyc)*(PKM2T-PKM2)/(Jpkm + PKM2T-PKM2)−kipkm*PKM2/(Jpkm + PKM2)
# steady state functions
# mTOR represents the active form of mTOR
mTOR = mTORT*GK(kamr + kamr’*KRAS + kamr”*PKM2,kimr + kimr’*CHL,Jmr,Jmr)
# cMyc represents the active form of cMyc
cMyc = cMycT*GK(kamyc + kamyc’*PKM2 + kamyc”*mTOR,kimyc,Jmyc,Jmyc)
# ‘Goldbeter-Koshand’ function (GK)
GB(arg1,arg2,arg3,arg4) = arg2-arg1+arg2*arg3+arg1*arg4
GK(arg1,arg2,arg3,arg4) = 2*arg1*arg4/(GB(arg1,arg2,arg3,arg4)+sqrt(GB(arg1,arg2,arg3,arg4)^2-4*(arg2-arg1)*arg1*arg4))
# parameters
# to simulate cancer: karas=1
# to simulate chloroquine treatment in cancer: karas=1, kimr’=1.5
# to simulate Vitamin C treatment in cancer: karas=1, kiras’=1.5
# to simulate PKM2 over-expression: karas=0.01, kapkm=0.5
# to simulate PKM2 depletion: karas=0.01, PKM2T=0.01
# to simulate cMyc over-expression: karas=1, cMycT=10
# to simulate cMyc depletion: karas=1, cMycT=0.1
p karas=0.01, karas’=0.1, kiras=0.1, kiras’=0, KRAST=1, Jkras=0.1
p kamr=0.01, kamr’=0.1, kamr”=2.25, kimr=0.1, kimr’=0, mTORT=1, Jmr=0.1
p kapkm=0.05, kapkm’=0.1, kapkm”=0.2, kipkm=0.2, PKM2T=1, Jpkm=0.1
p kamyc=0.01, kamyc’=0.2, kamyc”=0.2, kimyc=0.3, cMycT=1, Jmyc=0.1
p CHL=1, VitC=1
done
#2 CODE
# a model to generate mTOR–KRAS signal–response curves
# initial conditions
init KRAS=0, PKM2=0
# differential equations
# mTOR represents the active form of mTOR
mTOR’ = (kamr + kamr’*KRAS + kamr”*PKM2)*(mTORT-mTOR)/(Jmr + mTORT-mTOR)−(kimr + kimr’*CHL)*mTOR/(Jmr + mTOR)
# PKM2 represents the active form of PKM2
PKM2’ = (kapkm + kapkm’*KRAS + kapkm”*cMyc)*(PKM2T-PKM2)/(Jpkm + PKM2T-PKM2)−kipkm*PKM2/(Jpkm + PKM2)
# steady state functions
# KRAS represents the active form of KRAS
KRAS = KRAST*GK(karas + karas’*PKM2,kiras + kiras’*VitC,Jkras,Jkras)
# cMyc represents the active form of cMyc
cMyc = cMycT*GK(kamyc + kamyc’*PKM2 + kamyc”*mTOR,kimyc,Jmyc,Jmyc)
# ‘Goldbeter-Koshand’ function (GK)
GB(arg1,arg2,arg3,arg4) = arg2-arg1+arg2*arg3+arg1*arg4
GK(arg1,arg2,arg3,arg4) = 2*arg1*arg4/(GB(arg1,arg2,arg3,arg4)+sqrt(GB(arg1,arg2,arg3,arg4)^2-4*(arg2-arg1)*arg1*arg4))
# parameters
# to simulate cancer: karas=1
# to simulate chloroquine treatment in cancer: karas=1, kimr’=1.5
# to simulate Vitamin C treatment in cancer: karas=1, kiras’=1.5
# to simulate PKM2 over-expression: karas=0.01, kapkm=0.5
# to simulate PKM2 depletion: karas=0.01, PKM2T=0.01
# to simulate cMyc over-expression: karas=1, cMycT=10
# to simulate cMyc depletion: karas=1, cMycT=0.1
p karas=0.01, karas’=0.1, kiras=0.1, kiras’=0, KRAST=1, Jkras=0.1
p kamr=0.01, kamr’=0.1, kamr”=2.25, kimr=0.2, kimr’=0, mTORT=1, Jmr=0.1
p kapkm=0.05, kapkm’=0.1, kapkm”=0.2, kipkm=0.2, PKM2T=1, Jpkm=0.1
p kamyc=0.01, kamyc’=0.2, kamyc”=0.2, kimyc=0.3, cMycT=1, Jmyc=0.1
p CHL=1, VitC=1
done

The code for time course simulations

#3 CODE
# a model to simulate time courses
# initial conditions,
init KRAS= 0.0146, mTOR= 0.5365, PKM2= 0.0405, cMyc= 0.0607, AUTA= 0.0332, GLUT1= 0.1612
# differential equations
# KRAS represents the active form of KRAS
KRAS’= (karas + karas’*PKM2)*(KRAST-KRAS)/(Jkras + KRAST-KRAS)−(kiras + kiras’*VitC)*KRAS/(Jkras + KRAS)
# mTOR represents the active form of mTOR
mTOR’ = (kamr + kamr’*KRAS + kamr”*PKM2)*(mTORT-mTOR)/(Jmr + mTORT-mTOR)−(kimr + kimr’*CHL)*mTOR/(Jmr + mTOR)
# PKM2 represents the active form of PKM2
PKM2’ = (kapkm + kapkm’*KRAS + kapkm”*cMyc)*(PKM2T-PKM2)/(Jpkm + PKM2T-PKM2)−kipkm*PKM2/(Jpkm + PKM2)
# cMyc represents the active form of cMyc
cMyc’ = (kamyc + kamyc’*PKM2 + kamyc”*mTOR)*(cMycT-cMyc)/(Jmyc + cMycT-cMyc)−kimyc*cMyc/(Jmyc + cMyc)
# AUTA represents the active autophagy
AUTA’ = (kaau + kaau’*KRAS)*(AUTAT-AUTA)/(Jau + AUTAT -AUTA)−(kiau + kiau’*mTOR)*AUTA/(Jau + AUTA)
# represents the active form of GLUT1
GLUT1’ =(kaglu + kaglu’*cMyc)*(1-GLUT1)/(Jglu + 1-GLUT1)−kiglu*GLUT1/(Jglu + 1-GLUT1)
# parameters
# to simulate cancer: karas=1
# to simulate chloroquine treatment in cancer: karas=1, kimr’=1, AUTAT=0.1
# to simulate Vitamin C treatment in cancer: karas=1, kiras’=1
# to simulate PKM2 over-expression: karas=0.01, kapkm=0.5
# to simulate PKM2 depletion: karas=0.01, PKM2T=0.01
# to simulate cMyc over-expression: karas=1, cMycT=10
# to simulate cMyc depletion: karas=1, cMycT=0.1
p karas=0.01, karas’=0.1, kiras=0.1, kiras’=0, KRAST=1, Jkras=0.1
p kamr=0.01, kamr’=0.1, kamr”=2.25, kimr=0.1, kimr’=0, mTORT=1, Jmr=0.1
p kapkm=0.05, kapkm’=0.1, kapkm”=0.2, kipkm=0.2, PKM2T=1, Jpkm=0.1
p kamyc=0.01, kamyc’=0.2, kamyc”=0.2, kimyc=0.3, cMycT=1, Jmyc=0.1
p kaau=1, kaau’=7.5 kiau=0.01, kiau’=7.5, Jau=0. 1,
p kaglu=0.01, kaglu’=3, kiglu=1, Jglu=0.01
p CHL=1, VitC=1
done
